# The impact of breast cancer awareness campaigns on the knowledge, attitudes, and practices of breast cancer screening among Saudi female employees

**DOI:** 10.1371/journal.pone.0331765

**Published:** 2025-09-05

**Authors:** Nada A. Alomairy

**Affiliations:** Diagnostic Radiography Technology Department, Jazan University, Jazan, Saudi Arabia; Jazan University, SAUDI ARABIA

## Abstract

**Background:**

Breast cancer (BC) stands as a significant and prevalent malignancy impacting women. The increase in statistics is primarily due to delayed detection, often attributed to a lack of awareness of symptoms. Additionally, emotional barriers and unfavourable attitudes toward breast screening contribute to the escalating prevalence of BC. This study targeted female university employees due to their dual role as professionals and caregivers, with a high potential for personal and community impact. This study aimed to evaluate the effectiveness of the university’s breast cancer (BC) health campaign related to the knowledge, attitudes, and practices (KAP) of female employees.

**Materials and methods:**

A cross-sectional descriptive study was conducted in October and November 2023, following a BC awareness campaign. An online self-administered questionnaire was used to collect the data. The campaign, in the form of awareness, included posters, brochures, and lectures. The subjects were 290 female workers recruited from various departments of the university. Validation of the sampling and questionnaire, using a pilot test and reliability analysis, was conducted. Demographic data was analysed using frequency and percentage. Independent t-tests and one-way ANOVA were used to analyse the differences in knowledge and attitude towards breast cancer between various demographic variables.

**Results:**

The results revealed that the health campaign significantly increased knowledge and fostered positive attitudes toward BC. Participants who had previously attended any prior BC health campaign scored higher in both knowledge (7.45 ± 0.98) and attitude (5.30 ± 1.48) compared to those who had never attended (knowledge: 6.10 ± 1.10, attitude: 4.10 ± 1.41). There were significant differences in the knowledge scores based on education level (p = 0.044), with participants with higher education having a higher mean knowledge score (7.50 ± 0.85) compared to those with a bachelor’s degree (6.70 ± 1.02). Participants with a family history of breast cancer had similar knowledge scores to those without but lower attitude scores. Independent t-tests and one-way ANOVA indicated that education level and attendance at the breast cancer health campaign were the significant factors associated with knowledge and attitude scores.

**Conclusion:**

The findings show that there was a positive attitude towards BC. Nevertheless, additional interventions, such as counselling services and promoting available BC screening, must be added to facilitate screening behaviour. Future campaigns are also recommended to use diverse, culturally sensitive strategies targeting both younger and older women, as well as men.

## 1. Introduction

Breast cancer (BC) is one of the most serious and common cancers affecting women worldwide. It develops when cells in the breast grow out of control and form a tumour. Symptoms include a lump in the breast, a change in breast size, breast pain and fluid discharge from the nipple. According to the World Health Organization (WHO), BC is the most frequently diagnosed cancer worldwide, accounting for roughly 25% of all cancer cases in women. BC was the major cause of cancer-related mortality among women in 2020, with an expected 2.3 million new cases and 685,000 deaths [1–3]. The chance of having BC is influenced by a variety of factors including age, lifestyle, family history, and genetic susceptibility. Furthermore, race and socioeconomic status, especially education level, have been proven to influence both disease incidence and the survival rate [4].

When considering these global trends, it is important to observe that Saudi Arabia has witnessed a significant rise in the incidence and mortality rates of BC among women [5]. BC is currently the most common cancer in Saudi Arabia, mirroring the global rise in incidence. Recent studies have reported an increase in the cumulative risk of BC among females in Saudi Arabia, rising from 11.8 to 29.7 per 100,000 population between 2001 and 2017 [6]. A study by Alqahtani et al. (2020) reported that the incidence rate of BC was 14.8% for females and 8.5% for males in Saudi Arabia, with females having a cumulative risk of 0.81% [7]. Additionally, the Saudi Cancer Registry at King Faisal Specialist Hospital and Research Center reported approximately 930 new cases of BC detected annually in the country [8]. These rising statistics are attributed to factors such as delayed diagnosis at the advanced stage, which is highly related to a lack of knowledge about the symptoms, effective barriers and negative attitudes toward breast screening, all of which contribute to the rising disease burden.

BC is largely preventable, with early diagnosis being the most effective method of preventing its impacts. Effective screening modalities such as breast self-examination (BSE), clinical breast examination (CBE), and mammography play significant roles in early detection and improved treatment outcomes [9]. However, numerous studies conducted in Saudi Arabia [5,10–14] have indicated that while knowledge of BC and BSE is widespread, there remains a significant gap in screening practices [15].

These gaps are often influenced by cultural, emotional, and logistical barriers, which prevent many women from participating in screening programs. These barriers include negative perceptions about breast screening, fear of the procedure, lack of access to screening facilities, and cultural beliefs. In Saudi Arabia, religious considerations, family dynamics, and cultural beliefs all play a significant role in shaping BC awareness and screening behaviour among Saudi women. Cultural beliefs about gender roles and modesty may hinder women from visiting medical facilities for screenings that require physical examinations. In some cases, women may feel uncomfortable discussing their breast health due to cultural taboos, leading to delayed diagnoses. Family dynamics also play a role, with some women prioritising family needs over personal health concerns, which can impact their willingness to access screening or treatment. Moreover, religious beliefs also play a dual role—while some interpretations support self-treatment and disease prevention, others may lead to a fatalistic approach in which disease is seen as destiny, reducing health promotion activities [16,17].

In addition to religious and cultural barriers, socio-economic factors relating to work and time constraints play a role in the behaviour of Saudi women regarding breast cancer (BC) screening. Despite the fact that healthcare services, including screening, are provided free and are generally accessible, many women may find it difficult to reconcile their work schedules with the time required for physicians’ visits and screening. Reducing these cultural, religious, and socio-economic barriers aspects is vital for improving awareness and encouraging participation in BC screening programs. These gaps emphasise the need for increased public health measures, particularly awareness health campaigns that educate women about the importance of early detection, reducing the fear and misconceptions associated with screenings, and increasing accessibility to screening services, such as mobile mammography units. BC awareness initiatives, such as those that provide mobile mammography services, play an important role in boosting screening accessibility and promoting healthy behaviours [18].

To evaluate the effectiveness of such health campaigns, it is essential to evaluate women’s knowledge, attitudes and practices towards BC. Additionally, recognising the barriers to screening as well as the reasons for not participating in programs related to BC prevention would also assist in devising more focused interventions. The aim of this study is to examine the effect of BC awareness programs on Saudi women regarding their knowledge, attitudes and practices related to the disease, while also examining the perceived reasons for low participation in the current screening practices. This research employed a cross-sectional survey design to assess the influence of BC awareness health campaigns among Saudi female employees *after* the awareness campaign, using a structured questionnaire to collect the appropriate data from a diverse sample. Statistical analysis was conducted to identify trends and correlations between awareness levels and screening practices. By focusing on Saudi female employees, the research aimed to bridge the gap between knowledge and screening practices using this kind of health campaign. Furthermore, the study compared the knowledge and practices of participants who had attended any previous BC health campaigns with those who had not to explore potential differences in their awareness and screening practices. This study will guide relevant agencies, policymakers, academicians and associations concerned with the eradication of BC in Saudi Arabia to assess the BC campaign itself, the content they display, and how they provide knowledge for probable modification. The findings of this study are intended to provide information that will outline and improve learning and awareness programs on this hot issue.

## 2. Materials and methods

### 2.1. Study design

This non-randomised, cross-sectional descriptive study was conducted from October to November 2024 to assess (KAP) towards BC among women following a targeted BC awareness health campaign. The health campaign was implemented across seven colleges within a major university in Saudi Arabia. The colleges were chosen to ensure a wide demographic sample by representing a diverse range of female employees in terms of age and education level. The health campaign was mainly aimed at educating the community about the existence of BC as a public health issue and the importance of early detection through screening programs.

The campaign was delivered through a combination of printed materials (posters and brochures) and lectures/seminars by healthcare professionals. The printed resources were distributed in the seven colleges to provide general knowledge about breast cancer, while the 1.5-hour lectures and seminars held across seven university colleges offered a platform for detailed learning and interaction where the learners could question and engage with experts directly. The training covered basic subjects such as breast cancer risk factors, early detection techniques (BSE, CBE, and mammography), and periodic screening. This multi-mode delivery was designed to reach the participants with varying preferences for receiving information. The visual and auditory elements of the materials helped to inform the public of BC’s risk factors, its signs and symptoms, as well as the relevance of mammography and BSE. It was hoped that this would lead to the encouragement of regular screening as well as healthier lifestyles.

### 2.2. Participants

The participants in this study were selected through convenience sampling, which is a non-random sampling technique where participants are chosen based on their availability and willingness to participate. This sampling technique could limit the generalisability of the findings to the overall population. Despite this, efforts were made to enable participant diversity by involving workers from various colleges, education levels, and age groups. This ensured that the sample was representative of a wide population in the university setting. Based on Sharma et al.’s (2020) [19] sample size calculator, sufficient data to generalise this research outcome is N = 310 (with 95% confidence interval and 5% margin error). To meet this target, female employees from seven affiliated colleges were invited to participate. Female employees were approached with the help of the administration directors of the respective colleges. A total of 290 participants completed the questionnaire, yielding a response rate of 93.5%. For a variety of reasons, including time constraints, the remaining 20 participants either dropped out of the study or did not finish the survey. There was no replacement for these non-respondents.

### 2.3. Ethical consideration

To ensure transparency, all ethical considerations were adhered to, including obtaining approval from the Standing Committee Publication and Research Ethics- Jazan University (Reference number: REC-45/11/1118). Informed consent was acquired from all participants prior to their participation. This study was conducted in accordance with the ethical guidelines of the Declaration of Helsinki.

### 2.4. Data collection tool and scoring

All methods were carried out in accordance with the relevant guidelines and regulations. After the awareness campaign, the self-administered questionnaire was designed and validated after reviewing previous studies [9,20–23], ensuring its suitability for this investigation. The questionnaire was created first in English and then translated into Arabic. One linguistic expert translated the English version into Arabic, and another translated it back into English to ensure the questionnaire’s accuracy and consistency. To further validate the clarity of the questions and the survey design, a pilot test was conducted with 20 female employees from a different population to assess the comprehensibility and relevance of the items. The pilot test approach was used to assess the practical application and feasibility of the questionnaire in a small group before being administered to the full sample. To evaluate the knowledge and attitude portions of the study, the reliability coefficient was also determined through the Cronbach’s alpha test, and the values were 0.82 and 0.79, respectively. The results of this test reveal good internal consistency, therefore the questionnaire is appropriate for the objectives of this study.

The questionnaire was created using a Google form divided into four sections, with 26 questions in total. Section 1 was comprised of the demographic information (e.g., age and education level). Section 2 was about whether they had attended a breast cancer health campaign before and whether there was a family history of BC information. Section 3 was about the knowledge of BC after attending the campaign, which was comprised of BC symptoms such as perceived changes in the size of the nipples, pulling in of the nipples, abnormal pain, discharge from the nipples, redness and nipple rash. Section 4 enquired about the respondent’s attitude towards BC in light of the BC awareness campaign, which included topics such as whether BC can be prevented, whether self-examination is an effective examination method, the reasons for breast examination, female doctor preferences for examination, early detection methods and personal hygiene. For Section 3, we assessed the prompted knowledge by asking close-ended questions, with “Yes” answers scoring 1 point and “No” answers or “I don’t know” responses scoring 0 points. Attitude was assessed by asking close-ended questions, with correct answers scoring 1 point and incorrect answers or “I don’t know” responses scoring 0 points. The available score range for knowledge was 0–8 points, and for attitudes, it was 0–7 points, for a total of 0–15 points. Participants with scores greater than 70% of the maximum possible score in each category (i.e., above 5 for knowledge and above 4 for attitudes) were considered to have good knowledge and attitudes. Those who scored less than 70% were thought to have inadequate knowledge and attitudes. The 70% threshold for defining ‘good’ and ‘poor’ knowledge and attitudes was used because it has been widely employed in previous KAP research and is a standard cut-off used in public health studies to separate adequate and poor levels of knowledge in survey-based studies [24,25]. This cut-off percentage ensures that participants who are classified as having good knowledge and attitudes have demonstrated a sufficient level of awareness and understanding. The fifth section was about practices, which were measured by the frequency of BC examinations and the reasons for not doing so. This section included multiple-choice and open-ended questions.

The study was explained to potential participants via email, which included a brief overview of the research purpose, the voluntary nature of participation, the confidentiality of their responses, and a link to an online questionnaire created via Google Forms. Reminders were sent via WhatsApp. The respondents were assured of the confidentiality and anonymity of their responses by signing the informed consent on the first page of the questionnaire before their participation. This process ensured that they understood the nature of the study and their rights. After signing, they proceeded to complete the survey. The inclusion criteria for participation included female employees at the seven colleges who were 20 years of age or older, able to provide informed consent, and were willing to complete the questionnaire. The exclusion criteria included female employees who were not currently employed (e.g., on leave or sabbatical) or who declined to participate.

### 2.5. Data analysis

The study data was analysed using SPSS software version 13.0 using descriptive and univariate statistical methods. Descriptive statistics such as frequency, percentage, mean, and standard deviation were used to analyse the demographic data. Knowledge, attitude and practices in the context of BC health campaigns were first measured through frequency and percentage. Then, an independent t-test and one-way ANOVA were used to assess the differences in knowledge and attitude scores across the demographic variables, including age, education level, family history of breast cancer, and attendance at breast cancer awareness campaigns. The threshold for statistical significance was set at P < 0.05.

## 3. Results

Table 1 presents the demographic characteristics of the participants, their previous history of breast cancer screening, and their respective knowledge and attitude scores toward BC. The study involved 290 female participants. The majority of subjects (179, 62%) were in the 20–30 age category. More than half of the participants had a bachelor’s degree (177, 61%), and the remaining (113, 39%) had higher education qualifications beyond a bachelor’s degree. Only 62 (21%) reported that they had a family history of breast cancer, and 92 (32%) had attended a breast cancer campaign prior to the study campaign (e.g., Ministry of Health Pink October booths, hospital outreach days, etc.).

**Table 1:** Demographic characteristics of participants and their respective knowledge and attitude scores towards BC (N = 290).

Characteristic	N (%)	Knowledge Score (Mean ± SD)	p- value	Attitude Score (Mean ± SD)	p-value
**Age groups (years)**					
20–30	179 (62%)	7.10 ± 0.92	0.092	4.95 ± 1.41	0.145
31–40	79 (27%)	7.28 ± 1.04		5.30 ± 1.47	
41 and above	32 (11%)	6.12 ± 1.68		4.93 ± 1.43	
**Education**					
Bachelor	177 (61%)	6.70 ± 1.02	**0.044**	4.84 ± 1.47	0.120
Higher Education	113 (39%)	7.50 ± 0.85		5.34 ± 1.32	
**Family breast cancer history**					
Yes	62 (21%)	7.30 ± 0.90	0.705	4.81 ± 1.60	0.120
No	228 (79%)	7.10 ± 1.10		5.11 ± 1.38	
**Attending breast cancer campaign before**					
Yes	92 (32%)	7.45 ± 0.98	0.992	5.30 ± 1.48	**0.050**
No	198 (68%)	6.10 ± 1.10		4.10 ± 1.41	

In the study, the 31–40 age group had the highest mean knowledge score (7.28 ± 1.04) and attitude score (5.30 ± 1.47). Participants with a higher education had a higher mean knowledge score (7.50 ± 0.85) compared to those with a bachelor’s degree (6.20 ± 1.14), with a significant difference observed across education level (p = 0.044). Participants with a bachelor’s degree had the lowest mean attitude score (4.84 ± 1.47). Participants who had a family breast cancer history had a similar mean knowledge score to those who did not but a lower attitude score (4.81 ± 1.60). Lastly, participants who had attended a breast cancer health campaign before scored higher in both knowledge (7.45 ± 0.98) and attitude (5.30 ± 1.48) compared to those who had not. The results of the statistical analysis to define the independent variables (demographic and attending breast cancer health campaign characteristics) associated with the dependent factors (knowledge and attitude) show that, except for differences linked to education level and attending a breast cancer campaign, none of the differences are statistically significant.

Table 2 displays the distribution of the subjects’ knowledge levels gained after the university BC campaign. A significant portion of respondents (245, 84.5%) reported that changes in nipple size are a symptom of breast cancer, while 254 (87.6%) noted that a pulling in of the nipple signifies breast cancer. Additionally, 288 (99.3%) acknowledged that pain in the armpit could be indicative of breast cancer, whereas 37 (12.8%) disagreed that breast dimpling was a symptom. Moreover, 269 (92.8%) recognised nipple discharge as a potential sign of breast cancer. The respondents also identified breast lumps (238, 82.1%) and ruled out nipple rash (266, 91.7%) as symptoms. Lastly, 263 (90.7%) concurred that redness of the breast skin is a significant symptom of breast cancer.

**Table 2:** Knowledge about breast cancer in the light of BC health campaigns (N = 290).

Item	Categories	N (%)
Change in nipple size	Yes	245 (84.5%)
	No	45 (15.5%)
Pulling in nipple	Yes	254 (87.6%)
	No	36 (12.4%)
Pain in armpit	Yes	288 (99.3%)
	No	2 (0.7%)
Dimpling of breast skin	Yes	37 (12.8%)
	No	253 (87.2%)
Discharge from nipple	Yes	269 (92.8%)
	No	21 (7.2%)
Lump in the breast	Yes	238 (82.1%)
	No	52 (17.9%)
Nipple rash	Yes	266 (91.7%)
	No	24 (8.3%)
Redness in breast skin	Yes	263 (90.7%)
	No	27 (9.3%)

Table 3 illustrates the distribution of respondents regarding their attitudes towards BC after the university BC campaign. A high percentage of participants (245, 84.5%) reported that every woman was susceptible to breast cancer. Conversely, 211 (72.8%) disagreed that breast cancer is treatable. Additionally, 239 (82.4%) women indicated agreement that self-examination of the breast could not detect abnormalities, and 216 (74.5%) stated that they did not have any reason for examining their breasts. Moreover, 260 (89%) disagreed that early diagnosis influences treatment, while 162 (55.9%) agreed that personal hygiene might prevent breast cancer. Finally, 261 (90%) agreed that early diagnosis and treatment ensured a prolonged life.

**Table 3:** Attitude about breast cancer in the light of BC health campaigns (N = 290).

Item	Categories	N (%)
Every woman is at risk of breast cancer	Yes	245 (84.5%)
	No	45 (15.5%)
Breast cancer can be cured	Yes	79 (27.2%)
	No	211 (72.8%)
Self-examination cannot detect abnormalities	Yes	239 (82.4%)
	No	51 (17.6%)
If there are no symptoms, there is no need for breast	Yes	216 (74.5%)
	No	74 (25.5%)
examination Early diagnose does not influence treatment	Yes	260 (89.7%)
	No	30 (10.3%)
Personal hygiene prevents breast cancer	Yes	162 (55.9%)
	No	128 (44.1%)
Early diagnosis guarantees the prolonged life	Yes	261 (90.0%)
	No	29 (10.0%)

Table 4 shows the practices concerning breast cancer prevention. The majority of women (177, 61.0%) reported conducting breast examinations once or twice a year, while 40 (13.8%) mentioned examining their breasts 3–5 times annually. Additionally, only 18 (6.2%) reported examining their breasts every one to two months, and 55 (19.0%) stated that they examined their breasts once a month. The results showed that few of the participants (52, 18%) had undergone breast cancer screening (BCS) or had performed breast self-examinations (BSE).

**Table 4:** Practice for BC (N = 290).

Item	Categories	N (%)
The recommended examination for an early breast cancer detection	Once in a month	55 (19.0%)
	Once in 2 months	18 (6.2%)
	3-5 times in a year	40 (13.8%)
	Once or twice a year	177 (61.0%)
Ever had BCS or perform BSE	Yes	52 (18%)
	No	238 (82%)

**Fig 1:**
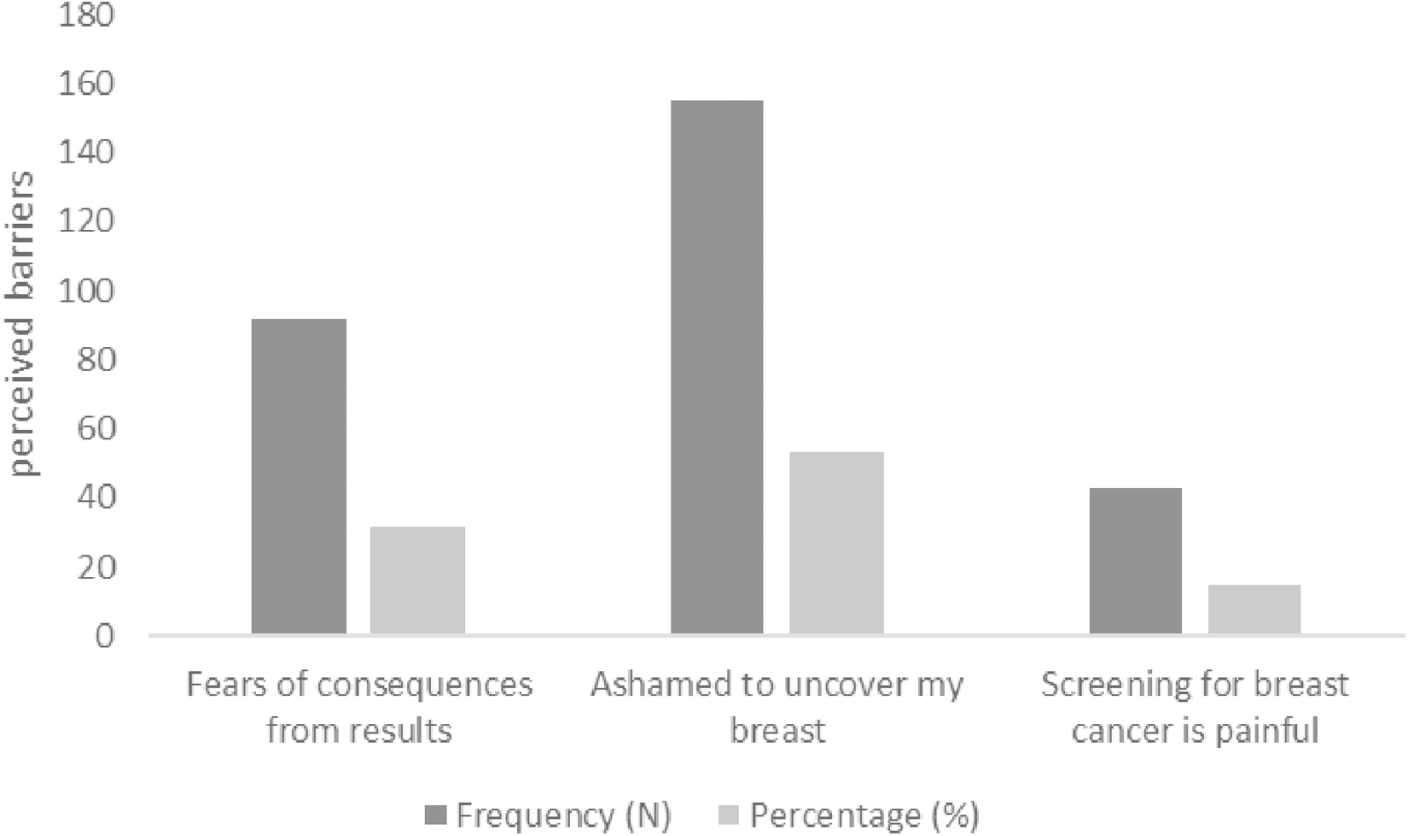
The perceived barriers (N = 290).

The perceived barriers mentioned by the women who had never screened for BC are demonstrated in Fig 1. The major factor that hindered BC was related to cultural and community barriers, including items related to shyness about being uncovered or touched by others (155, 53.45%), followed by fear of the consequences after examination (92, 31.72%) and the concern that both the breast screening and examination (mammograms) are painful (43, 14.83%).

## 4. Discussion

The significance of this study is manifold, given that KAP are crucial for the early detection and effective management of breast cancer. The women’s level of knowledge and attitude, along with their perceptions of preventive measures such as screening methods and knowing the reasons for not screening, are pivotal in preventing breast cancer in the general female population. Therefore, this research aimed to assess the impact of the university BC health campaign on KAP among female employees.

This study revealed a number of interesting findings about the knowledge, attitudes and practices towards BC among female employees in Saudi Arabia. The 31–40 age group exhibited the highest mean knowledge (7.28 ± 1.04) and attitude scores (5.30 ± 1.47), although the difference was not statistically significant compared to the 20–30 age group. In the present investigation, the educational level of female employees emerged as a significant determinant of knowledge, indicating that education plays a significant role in knowledge about the topic. This discovery aligns with findings from other studies [12,25,26], although it contrasts with the results of Agboola et al. (2009) [27]. In our current study, participants who had previously attended any BC awareness health campaigns had higher knowledge and attitude scores compared to those who did not, suggesting that engaging in such health campaigns significantly enhances attitudes toward BC.

Our research findings regarding breast cancer knowledge indicated that, out of the total respondents (n = 290), a significant majority were well informed about the symptoms of breast cancer. They mentioned attentively observing university health campaigns addressing this critical issue in Saudi Arabia, which poses a significant threat to women’s well-being. This is consistent with previous studies that have also found a high level of awareness regarding BC symptoms among female students and healthcare professionals [9,13,20,21,23]. There were misconceptions about breast dimpling as one of the symptoms of BC, indicating areas where further education may be beneficial.

The results of our study indicate that women generally have a positive attitude towards breast cancer, believing that every woman is susceptible to it. Early detection can enhance treatment efficacy, and personal hygiene practices can lower the risk of developing breast cancer. These results are comparable to those of a previous study [20]. Nevertheless, there were misunderstandings regarding the treatability of breast cancer, highlighting areas where additional education could prove beneficial.

In this study, the author observed that health campaign awareness efforts regarding breast cancer fall significantly short of expectations, and that discrepancies between knowledge, attitudes and practices regarding breast cancer among women in Saudi Arabia were observed. The BC health campaign delivered the right knowledge to the women, establishing a positive attitude towards the treatment and preventive mechanisms to keep them safe from breast cancer but the results were less efficient regarding BC practice, where only 18% of the studied university employee participants had ever performed BSE and screened for BC, even though the healthcare services are provided free of charge to the population, compared to 16.2% observed in Abdel-Aziz et al. (2018) [12]. A lack of understanding of how to conduct BSE was highlighted as the primary reason for not practicing it. This study’s results align closely with those of Mbiereagu and Etumnu (2020) [18].

Women who were less inclined to participate in BC preventive screenings mentioned reasons such as socio-cultural barriers, including shyness about being uncovered or touched by others, fear of receiving a cancer diagnosis, and pain from the breast screening (mammogram) examination. This observation is consistent with the findings of previous studies [12,20,22,28,29], which also identified discrepancies between knowledge, attitudes and practices regarding BC among women in Saudi Arabia.

Addressing these barriers requires targeted practical interventions. The provision of culturally appropriate counselling services and using female health workers in screening centres to alleviate privacy and discomfort issues can eliminate these. The development of educational videos and workshops demonstrating proper BSE procedures can also reduce the practice gap. To further improve BC awareness and screening behaviours, public health and media health campaigns should be diverse and culturally appropriate, targeting both younger and older women, as well as men. Utilising platforms such as television, radio, newspapers, magazines, pamphlets, and SMS messages can effectively enhance the awareness of breast cancer screening (BCS). We suggest integrating breast self-examination (BSE) into the curriculum of schools and universities for females and organising community health campaigns at shopping malls and various public gatherings. These initiatives aim to enhance knowledge and encourage practice, ultimately reducing the morbidity and mortality rates associated with breast cancer.

## 5. Study limitations and future work

This study has several limitations that should be considered when interpreting the results. First, the results may not be applicable to all regions of Saudi Arabia, as the study involved only women from the southern region. Second, the study utilised a cross-sectional design which introduced the potential for recall bias and social desirability bias. Third, there is a lack of a qualitative component, which could have provided more in-depth insights into personally perceived barriers, particularly those related to sociocultural factors. Further investigation into cultural values and beliefs is necessary to gain a deeper understanding of the factors influencing women’s awareness of BCS and BSE. Future studies should also focus on assessing the effectiveness of specific interventions, such as formal training programs for BSE and mobile mammography services, in order to determine their impact on screening behaviours. Additionally, future studies would be enhanced by the addition of a pre-test to establish a baseline and more accurately assess the impact of the campaign on knowledge and attitudes over time. Advanced psychometric validation methods such as Exploratory Factor Analysis (EFA) and Confirmatory Factor Analysis (CFA) should be used to assess the construct validity of the questionnaire.

## 6. Conclusion

The study revealed that the participants demonstrated high knowledge and awareness regarding BC symptoms and risk factors. However, the results indicate a low level of participation in BSE and BC screening, which are perceived as less valuable tools in conservative societies such as Saudi Arabia, thus affecting the BC prevention efforts. These findings emphasise the need for interventions that not only increase awareness but also address the behavioural and societal determinants of screening behaviour. The implementation of culturally sensitive learning methods and counselling services can also improve BC prevention and encourage women to adopt proactive health practices.

## Supporting information

S1 File Questionnaire form used in the study. (PDF)
