# Supplementary material for: The impact of breast cancer awareness campaigns on the knowledge, attitudes, and practices of breast cancer screening among Saudi female employees
Source: PLoS One. 2025 Sep 5;20(9):e0331765. doi: 10.1371/journal.pone.0331765 (PMC12412964; doi:10.1371/journal.pone.0331765)
Supplement: S1 File — (PDF) [file pone.0331765.s001.pdf]

Appendix

Questionnaire Form

I am an assistant professor in Jazan University, conducting a research study entitled "The Impact of Jazan University Breast Cancer Awareness Campaign on Female Students and Employees". The purpose of this research is to evaluate the knowledge, attitude, and practices regarding breast cancer among women post-campaign of Jazan University breast cancer 2021. Your participating will involve answering a few questions in a questionnaire and should only take about 5 minutes. Do not worry about picking the wrong answer, we just want to see what misconceptions and common beliefs.

Your involvement in the study is voluntary and your name is not required on the questionnaire. Your response will be anonymous and will never linked to your personality. The results of the research study may be published, but your personal information will not be used. Your identity will not be associated with your responses in any published format. The findings from this project will provide information that will outline and improve learning and awareness program on this hot issue with no cost to you other than the time it takes for the survey. If you have any questions about this research project, please feel free to email me at alumairi@jazanu.edu.sa. Thank you for your cooperation.

Section 1: Demographic Information

1- Age

- o 20-30
- o 31-40
- o >40

2- Level of education:

- o Diploma or less
- o BS
- o Masters
- o PhD

3- Do you have a family breast cancer history?

- o Yes
- o No

**4**	**Have you ever undertaken Breast cancer screening?**	o No	o yes
**5**	**Do you think campaigns provide proper knowledge about breast cancer?**	o No	o yes
**6**	**Do you think these campaigns should be continuing?**	o No	o yes

<u>Section 2:</u> Knowledge regarding breast cancer after breast cancer campaign
Answer "yes" or "No" to the following breast cancer symptoms

**8**	**Change in nipple size**	o No	o yes
**9**	**Pulling in nipple**	o No	o yes
**10**	**Pain in armpit**	o No	o yes
**11**	**Dimpling of breast skin**	o No	o yes
**12**	**Discharge from nipple**	o No	o yes

**13**	**Lump in the breast**	○ No	○ yes
**14**	**Nipple rash**	○ No	○ yes
**15**	**Redness in breast skin**	○ No	○ yes

Section 3: Attitude regarding breast cancer after breast cancer campaign

Answer "yes" or "No" to the following

**16**	Every woman is at risk of breast cancer	○ No	○ yes
**17**	Breast cancer can be cured	○ No	○ yes
**18**	Self-examination cannot detect abnormalities	○ No	○ yes
**19**	I found no reason to examine my breast	○ No	○ yes
**20**	Would do self-examination if I came to know about benefits	○ No	○ yes
**21**	Women prefer female doctors for breast examination	○ No	○ yes
**22**	If there are no symptoms, there is no need for breast examination	○ No	○ yes
**23**	Early diagnose does not influence treatment	○ No	○ yes

**24**	Personal hygiene prevents breast cancer	○ No	○ yes
**25**	Early diagnosis guarantees the prolonged life	○ No	○ yes

Section 4: Breast cancer prevention practices

Please choose the suitable answer

26- The recommended examination for an early breast cancer detection

- Once in a month
- Once in 2 months
- 3-5 times in a year
- Once or twice a year

27- Reason for not screeing breast cancer

- fears of consequences results
- A shamed to uncover my breast
- screening for breast cancer is painful
